# Supplementary material for: Content-rich biological network constructed by mining PubMed abstracts
Source: BMC Bioinformatics. 2004 Oct 8;5:147. doi: 10.1186/1471-2105-5-147 (PMC528731; doi:10.1186/1471-2105-5-147)
Supplement: Additional File 2 — The original results of the above study (non-essential files are deleted to keep the file size under the limit set by BMC bioinformatics). [file 1471-2105-5-147-S2.bz2 › chilibotAdditionalFile2/dip05/670ID11110785E11072/html/CCR3_CXCL11.html]

 


 **CCR3** and **CXCL11** 
  
Found 10 abstracts in PubMed, retrieved 05.  
 

 What does Google say? 
 PDF only 
| .edu only 

---

**Interactive relationship** (e.g. stimulation, inhibition, etc)

**Inhibitory relationship**- We alsodemonstrate that the CXCR3 ligands inhibit  **CCR3**  mediated functional responses of both human eosinophils and  **CCR3**  transfectants induced by all three eotaxins, with  **CXCL11**  being the most efficacious antagonist.  Ref: 12884299 Eur J Immunol, 2003
**Neutral relationship**- **CCR3**  functional responses are regulated by both CXCR3 and its ligands CXCL9, CXCL10 and  **CXCL11** .  Ref: 12884299 Eur J Immunol, 2003

**Non-interactive relationship** (e.g. studied together, co-existance, homology, etc.)

- In contrast, CCL18 and I TAC  [ **CXCL11** ]  interferon inducible T cell alpha chemoattractant  [ **CXCL11** ]  were neutral antagonists, inhibiting eotaxin induced 35S GTPgammaS binding, with minimal effect on basal coupling of  **CCR3**  to G proteins.  Ref: 12450563 Eur J Pharmacol, 2002
- The examination of  **CCR3**  CCR1 chimeric constructs revealed that CCL11 and  **CXCL11**  share overlapping binding sites contained within the  **CCR3**  extracellular loops, a region that was previously shown to be essential for effective receptor activation.  Ref: 12884299 Eur J Immunol, 2003
